# A New Smartphone-Based Cognitive Screening Battery for Multiple Sclerosis (icognition): Validation Study

**DOI:** 10.2196/53503

**Published:** 2025-01-20

**Authors:** Stijn Denissen, Delphine Van Laethem, Johan Baijot, Lars Costers, Annabel Descamps, Ann Van Remoortel, Annick Van Merhaegen-Wieleman, Marie D’hooghe, Miguel D'Haeseleer, Dirk Smeets, Diana M Sima, Jeroen Van Schependom, Guy Nagels

**Affiliations:** 1 AIMS Lab Center for Neurosciences Universitair Ziekenhuis Brussel, Vrije Universiteit Brussel Brussels Belgium; 2 icometrix Leuven Belgium; 3 Department of Physical and Rehabilitation Medicine Universitair Ziekenhuis Brussel Brussels Belgium; 4 Neurology Department National Multiple Sclerosis Center Melsbroek Belgium; 5 Neurology Department Universitair Ziekenhuis Brussel Brussels Belgium; 6 Center for Neurosciences Vrije Universiteit Brussel Brussels Belgium; 7 Department of Electronics and Informatics Vrije Universiteit Brussel Brussels Belgium; 8 St Edmund Hall University of Oxford Oxford United Kingdom

**Keywords:** multiple sclerosis, telemedicine, cognition, memory, information processing speed, mobile phone

## Abstract

**Background:**

Cognitive deterioration is common in multiple sclerosis (MS) and requires regular follow-up. Currently, cognitive status is measured in clinical practice using paper-and-pencil tests, which are both time-consuming and costly. Remote monitoring of cognitive status could offer a solution because previous studies on telemedicine tools have proved its feasibility and acceptance among people with MS. However, existing smartphone-based apps include designs that are prone to motor interference and focus primarily on information processing speed, although memory is also commonly affected.

**Objective:**

This study aims to validate a smartphone-based cognitive screening battery, icognition, to detect deterioration in both memory and information processing speed.

**Methods:**

The icognition screening battery consists of 3 tests: the Symbol Test for information processing speed, the Dot Test for visuospatial short-term memory and learning, and the visual Backward Digit Span (vBDS) for working memory. These tests are based on validated paper-and-pencil tests: the Symbol Digit Modalities Test, the 10/36 Spatial Recall Test, and the auditory Backward Digit Span, respectively. To establish the validity of icognition, 101 people with MS and 82 healthy participants completed all tests. Of the 82 healthy participants, 20 (24%) repeated testing 2 to 3 weeks later. For each icognition test, validity was established by the correlation with its paper-and-pencil equivalent (concurrent validity), the correlation and intraclass correlation coefficient (ICC) between baseline and follow-up testing (test-retest reliability), the difference between people with MS and healthy participants, and the correlation with other clinical parameters such as the Expanded Disability Status Scale.

**Results:**

All icognition tests correlated well with their paper-and-pencil equivalents (Symbol Test: *r*=0.67; *P*<.001; Dot Test: *r*=0.31; *P*=.002; vBDS: *r*=0.69; *P*<.001), negatively correlated with the Expanded Disability Status Scale (Symbol Test: ρ=–0.34; *P*<.001; Dot Test: ρ=−0.32; *P*=.003; vBDS: ρ=−0.21; *P*=.04), and showed moderate test-retest reliability (Symbol Test: ICC=0.74; *r*=0.85; *P*<.001; Dot Test: ICC=0.71; *r*=0.74; *P*<.001; vBDS: ICC=0.72; *r*=0.83; *P*<.001). Test performance was comparable between people with MS and healthy participants for all cognitive tests, both in icognition (Symbol Test: *U*=4431; *P*=.42; Dot Test: *U*=3516; *P*=.32; vBDS: *U*=3708; *P*=.27) and the gold standard paper-and-pencil tests (Symbol Digit Modalities Test: *U*=4060.5, *P*=.82; 10/36 Spatial Recall Test: *U*=3934; *P*=.74; auditory Backward Digit Span: *U*=3824.5, *P*=.37).

**Conclusions:**

icognition is a valid tool to remotely screen cognitive performance in people with MS. It is planned to be included in a digital health platform that includes volumetric brain analysis and patient-reported outcome measures. Future research should establish the usability and psychometric properties of icognition in a remote setting.

## Introduction

### Background

Medicine is increasingly digitalizing, and there are compelling reasons to stimulate this trend. Clinicians can more easily access and share electronic health records, and storing data in a digital format facilitates visualization and organization in research databases, yielding new insights into pathology and disease management. Moreover, electronic health records drive artificial intelligence research [1], while artificial intelligence, in turn, further stimulates storing records digitally [2], closing a positive feedback loop. Far from being a mere “nice to have,” digital medicine was crucial during the COVID-19 pandemic [3], enabling telemedicine services to be provided when social distancing was essential.

Telemedicine provides practical solutions for people with multiple sclerosis (MS), a chronic disease characterized by inflammation and degeneration of the central nervous system [4]. Telemedicine tools are well accepted by patients, and their feasibility and cost-effectiveness have been established previously [5]. Moreover, patients tend to objectively benefit from the use of these tools, which can, for example, aid in fatigue management [6] and improve cognitive function [7]. The latter is important because nearly half of the people with MS have cognitive impairment [8], which has significant repercussions on daily life activities, societal participation, employment, and susceptibility to psychiatric disorders [9].

Telemedicine could also facilitate cognitive monitoring of people with MS. First, it would allow increasing temporal resolution of cognitive trajectories because cognitive tests are usually performed during routine follow-up sessions that are months apart. This is problematic because cognitive decline can be sudden, unexpected, and severe (eg, in case of a relapse) [10,11]. Remote testing could help detect minimal changes early, enabling timely intervention. Second, it would unburden clinicians because current practice relies on paper-and-pencil tests administered under the supervision of a trained examiner.

Smartphones especially provide a window of opportunity, with an estimated 6.7 billion subscriptions worldwide (69% of the population) [12]. However, current smartphone-based cognitive assessments focus primarily on information processing speed (IPS) [13]; yet, besides slowed IPS, the hallmark cognitive problem in MS is impaired memory [14], for which the first smartphone test was only recently introduced by Podda et al [15]. Memory assessments emerged earlier on tablet devices [16] but are less suitable for consistent follow-up because tablet devices are used far less frequently than smartphones. Furthermore, smartphone tests might be prone to motor interference. They are predominantly digital versions of the Symbol Digit Modalities Test (SDMT) [17], which is a popular test in clinical practice to measure IPS and has excellent psychometric properties [18]. Digitalizing the SDMT allows randomizing its key, which could reduce practice effect as reported in the study by Pereira et al [19]. However, creating an exact digital replica of the SDMT requires patients to choose from 9 small buttons on the screen, which could cause motor interference because fine motor skills are commonly affected in MS [20].

### Objectives

To tackle these limitations, in this study, we aim to validate a new smartphone-based cognitive screening battery called “icognition.” It is a quick, smartphone-based screening tool for remote follow-up of the 2 most commonly impaired cognitive domains in MS: IPS and memory [14]. It is intended to be part of the recently established icompanion app, a digital diary for people with MS [21]. Regular remote screening could enable faster confirmation of cognitive deterioration by a neuropsychologist and prompt intervention by the patient’s neurologist.

## Methods

### Study Design

This is an observational case-control study designed to validate the icognition smartphone app.

### Participants

Study participants were recruited between June 17, 2021, and January 3, 2023. People with MS were enrolled from the outpatient clinics of the neurology department at Universitair Ziekenhuis Brussel (secondary care) and the National MS Center at Melsbroek (tertiary care). They were recruited by the local study nurse during their follow-up visit. Healthy control participants were recruited via leaflets and the social networks of the researchers involved. Inclusion criteria for people with MS were a confirmed diagnosis of MS according to the McDonald criteria [22]. People with MS were excluded if they had been hospitalized for reasons other than rehabilitation or if they had experienced a relapse within the past month. Both people with MS and healthy control participants were excluded if they had any other neurological or psychiatric disorder or learning disorder. A total of 101 people with MS and 82 healthy control participants (matched on age, sex, and education level) met the inclusion and exclusion criteria for this study. All participants were either Dutch-speaking or bilingual, including Dutch, and were aged ≥18 years.

### Ethical Considerations

This study was approved by the medical ethics committee of Universitair Ziekenhuis Brussel (BUN 143201940335) and the National MS Center at Melsbroek. All participants signed informed consent (in Dutch) before inclusion. Data were pseudonymized and stored in the protected OneDrive cloud service (Microsoft Corporation) of Vrije Universiteit Brussel. When publicly shared via GitHub, as explained in the Data Availability section, the data will be anonymized. This manuscript presents the primary analysis of these data. Participants did not receive compensation for taking part in the study.

### icognition Screening Battery

**Figure 1:**
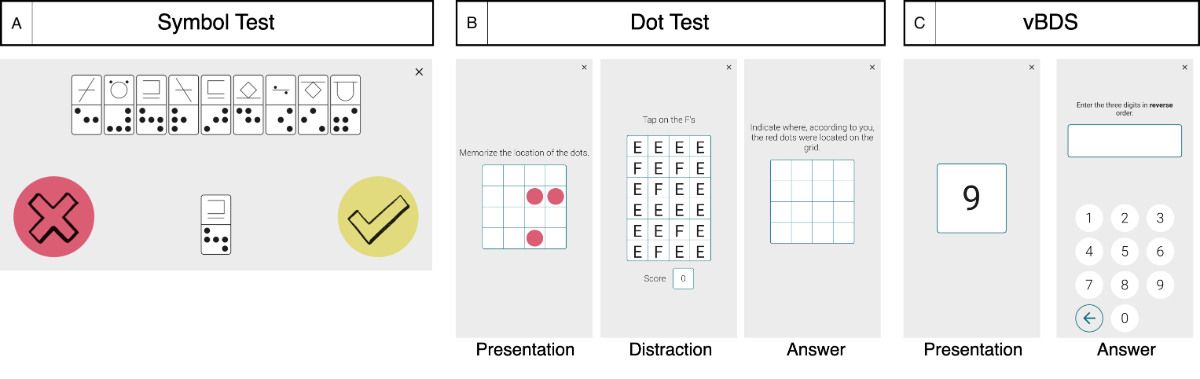
Screenshots of the icognition tests (although the instructions were in Dutch during testing, they are presented here in English). (A) The Symbol Test for information processing speed (score=number of correct responses [does the symbol combination occur in the key?] in 90 s). (B) The Dot Test for visuospatial short-term learning and memory (score=total number of correctly indicated dots across 10 trials [maximum score=30]). (C) Visual Backward Digit Span (vBDS) for working memory (score=sum of span lengths of correct spans [correct inversion of shown span]).

The icognition cognitive screening battery consists of 3 tests (Figure 1).

The Symbol Test is based on the computerized Digit Symbol Substitution Test presented in the study by Rypma et al [23]. In the Symbol Test, a combination of symbols is presented to the participants, one at a time. A key, consisting of 9 pairs of symbols, is displayed at the top and is shuffled for each trial. For each trial, the participant needs to indicate whether the presented combination appears in the key. The total score is the number of correct answers provided in 90 seconds. This test is designed to assess IPS.

The Dot Test consists of 3 phases. In the first phase, a participant is presented a 4×4 grid in which 3 dots are shown for 3 seconds. Next, as a distractor task, the participant is shown a 4×6 grid of “E” and “F” shapes and must identify as many “F” shapes as possible in 4 seconds. In the last phase, the participant must indicate in an empty 4×4 grid where the 3 dots of the first phase were located. The Dot Test is inspired by the Dot Memory Test presented in the study by Sliwinski et al [24], with all grids reduced in size compared to their version (5×5 grids). We also implemented a criterion for the distractor task, requiring the participant to identify at least 3 “F” shapes. If this criterion was not met, the trial was restarted. The 3 dots could not be aligned on 1 line or form an L-shape within a 2×2 block of cells. The total score is the number of correctly indicated dots across 10 trials. This test is designed to assess visuospatial short-term memory and learning.

In the visual Backward Digit Span (vBDS), a series of digits is presented on the screen one by one, each for 1 second, as described in the study by Hilbert et al [25]. The participant must then list the digits in reverse order. Spans were randomly generated with digits between 0 and 9, with the following constraints: a digit can appear only once in the span, and a chain of ≥3 digits cannot have a fixed increment or decrement of 1 or 2 digits, in accordance with Woods et al [26]. Scoring is based on the sum of all correct span lengths; for example, if a participant correctly recalls 2 spans of length 3 and 1 span of length 4, the total score is 10. This test is designed to assess working memory.

All tests were performed on a Samsung Galaxy A10 smartphone (6.2-inch screen size) and were supervised by a test examiner. Each test was directly preceded by a practice phase to familiarize the participants with the respective test. This phase consisted of 5 trials for the Symbol Test, 3 for the Dot Test, and 4 (2 of length 3 and 2 of length 4) for the vBDS. The Symbol Test is performed with the smartphone in landscape orientation, whereas for the Dot Test and vBDS, the smartphone must be in portrait orientation. In the design of icognition, careful consideration was given to the potential biasing influence of fine motor impairment in MS [20]. Motor interference was minimized by using 2 large buttons for the Symbol Test (in contrast to digital SDMT variants where participants must choose from 9 smaller buttons [27]) and not placing any restrictions on the response time in the other icognition tests.

### Validation Procedure

The procedure to validate icognition is based on the study by Benedict et al [28] and involves assessing 4 criteria, as outlined in the following subsections.

#### Concurrent Validity

We assessed how well each icognition test correlates with its paper-and-pencil equivalent. For the Symbol Test, the equivalent was the SDMT [17]. In the SDMT, a sheet is presented to the participant with a key of 9 symbol-digit pairs at the top and a list of symbols without corresponding digits. In 90 seconds, the participant must convert as many symbols to digits as possible from the list, reading them out loud to the examiner, using the key. The Dot Test is based on the 10/36 Spatial Recall Test (SPART) [29]. In the SPART, the participant is shown a 6×6 grid with a pattern of 10 dots for 10 seconds. Subsequently, the grid is removed, and the participant is asked to replicate the pattern using 10 checkers. This process is repeated 3 times. The final score is the total number of correctly placed checkers across all trials. Finally, for the vBDS, a modified version of the Wechsler Adult Intelligence Scale, Fourth Edition, auditory Backward Digit Span [30] was used. In the auditory Backward Digit Span, digit spans are read out loud to the participant, who is asked to repeat them in reverse order. The original test consists of 2 trials for each span length, starting with a span of 2 digits and increasing by 1 digit each time the participant correctly completes at least 1 of the 2 trials. As discussed in Woods et al [31], in the original Wechsler Adult Intelligence Scale, Fourth Edition, design, participants with the same score can have a different number of correct spans. To mitigate this, we used a fixed number of spans, ranging from 3 to 7 digits in length (Table S1 in Multimedia Appendix 1). The complete list was always administered for each participant. The scoring metric is the same as described earlier for the icognition Backward Digit Span.

#### Test-Retest Reliability

Benedict et al [28] mention that test-retest reliability should be assessed on a “small sample” of either people with MS or healthy controls [28]. We aimed to retest the healthy controls 2 to 3 weeks after baseline testing. We used the intraclass correlation coefficient (ICC) to assess agreement between baseline and retesting, which is explained in more detail in the Statistical Analyses subsection.

#### Comparison of Performance

For each test, we compared the performance of people with MS to that of age-, sex-, and education level–matched healthy controls using the Mann-Whitney *U* test. The analysis was then repeated after correcting test performance for age, sex, and education level (Figure S1 in Multimedia Appendix 1). Details of the correction methodology are presented in the Statistical Analyses subsection.

#### Assessment of Correlations

Finally, we assessed the correlation of each icognition test with the Expanded Disability Status Scale (EDSS) [32], disease duration, the Beck Depression Inventory (BDI) [33], the Fatigue Scale for Motor and Cognitive Functions (FSMC) [34], education level, and age.

### Data Curation

Data were entered independently by 2 researchers (SD and DVL). Conflicts in data entry were resolved through mutual discussion.

### Statistical Analyses

We used an α level of .05 for all analyses. Participants with missing data on a certain test were only excluded for that specific test. We used the Spearman correlation for nonlinear and categorical variables (EDSS, BDI, FSMC, and education level), whereas the Pearson correlation was used otherwise. Mann-Whitney *U* tests were used for between-group distribution comparisons.

To assess test-retest reliability, following the approach of van Oirschot et al [35] and the guidelines by Koo and Li [36], we used the following ICC type: “two-way mixed effects, absolute agreement, single rater/measurement” (ICC[A,1]). In this study, the smartphone app served as the sole “rater,” which is why we used a 2-way mixed effects model, appropriate when the “selected raters are the only raters of interest” [36]. For the same reason, the “type” of ICC was “single rater/measurement,” while for “definition,” we used “absolute agreement,” reflecting the extent to which 1 rater’s score (in our case, baseline testing) equals the other rater’s score (in our case, retesting). The decision process is illustrated in the decision flowchart in the study by Koo and Li [36]. To interpret the magnitude of the test-retest reliability, we used the guidelines by Koo and Li [36]: poor (<0.50), moderate (0.50-0.75), good (0.75-0.90), and excellent (>0.90).

The magnitude of correlations was interpreted using the classification by Portney and Watkins [37]: small (<0.25), fair (0.25-0.50), moderate to good (0.50-0.75), and excellent (>0.75).

Finally, for each cognitive test, we fitted a regression equation with age, sex, and education level as independent variables and test performance as dependent variable on the healthy control data. This allows calculation of the expected score of a participant, given their age, sex, and education level, and subsequently comparison with the actual score of the participant, resulting in a *z* score. The technical details of the procedure, including the required values for performing *z* normalization on other data, are presented in Multimedia Appendix 1, while Table S2 in Multimedia Appendix 1 presents the values necessary to perform the procedure. Test performance was considered impaired if the *z* score was ≤−1.5, based on the study by Benedict et al [38].

## Results

### Overview

Participant characteristics are presented in Table 1.

**Table 1:** Participant characteristics (n=183)^a^.

				People with MS^b^ (n=101)		Healthy controls (n=82)		*P* value
**Demographics**								
	**Age (y), mean (SD)**			45.4 (10.0)		46.8 (14.7)		.59^c^
	**Sex, n (%)**							.74^d^
		Female	63 (62.4)		54 (65.9)			
		Male	38 (37.6)		28 (34.1)			
	**Education, median (IQR)**			15 (12-17)		15 (13-17)		.74^c^
**MS specific**								
	**Disease duration (y), mean (SD)**			11.7 (7.4)		—^e^		—
	**MS type, n (%)**					—		—
		RRMS^f^	86 (85.1)					
		SPMS^g^	8 (7.9)					
		PPMS^h^	7 (6.9)					
	**EDSS^i^** **, median (IQR)**			3 (2-4)		—		—
**Paper-and-pencil tests**								
	**SDMT^j^** **, mean (SD)**			58.5 (10.0)		58.3 (9.9)		.82^c^
		Those considered impaired, n (%)	7 (6.9)		6 (7.3)		.99^d^	
	**10/36 SPART^k^** **, mean (SD)**			20.6 (4.3)		20.1 (4.6)		.74^c^
		Those considered impaired, n (%)	8 (7.9)		4 (4.9)		.60^d^	
	**Auditory BDS^l^** **, mean (SD)**			48.4 (18.7)		46.0 (17.6)		.37^c^
		Those considered impaired, n (%)	9 (8.9)		6 (7.3)		.90^d^	
	**BDI^m^** **, mean (SD)**			9.3 (5.7)		5.8 (5.1)		<.001^c^
	**FSMC^n^** **, mean (SD)**			58.7 (16.4)		39.9 (12.1)		<.001^c^
**icognition tests**								
	**Symbol Test, mean (SD)**			24.8 (6.3)		25.4 (6.4)		.42^c^
		Number impaired, n (%)	13 (12.9)		7 (8.5)		.49^d^	
	**Dot Test, mean (SD)**			21.8 (5.1)		21.2 (4.9)		.32^c^
		Number impaired, n (%)	9 (8.9)		7 (8.5)		.99^d^	
	**Visual BDS, mean (SD)**			46.9 (16.9)		43.8 (16.7)		.27^c^
		Number impaired, n (%)	8 (7.9)		9 (11)		.65^d^	

^a^The following variables had missing values: Expanded Disability Status Scale (n=7), 10/36 Spatial Recall Test (people with multiple sclerosis: n=1; healthy controls: n=1), Beck Depression Inventory (people with multiple sclerosis: n=2; healthy controls: n=1), Fatigue Scale for Motor and Cognitive Functions (people with multiple sclerosis: n=2; healthy controls: n=7), Dot Test (people with multiple sclerosis: n=3; healthy controls: n=1), and visual Backward Digit Span (people with multiple sclerosis: n=1). An additional 3 participants were excluded from the Dot Test analyses because they had been tested with an earlier version of icognition where a larger grid size was used. We reduced the grid size after these 3 participants were tested because we deemed this test to be too difficult. However, these 3 participants were included when the mean (SD) and number of participants considered impaired was calculated.

^b^MS: multiple sclerosis.

^c^Mann-Whitney *U* test.

^d^Chi-square test.

^e^Not applicable.

^f^RRMS: relapsing-remitting multiple sclerosis.

^g^SPMS: secondary progressive multiple sclerosis.

^h^PPMS: primary progressive multiple sclerosis.

^i^EDSS: Expanded Disability Status Scale

^j^SDMT: Symbol Digit Modalities Test.

^k^SPART: 10/36 Spatial Recall Test.

^l^BDS: Backward Digit Span

^m^BDI: Beck Depression Inventory.

^n^FSMC: Fatigue Scale for Motor and Cognitive Functions.

### Concurrent Validity

**Figure 2:**
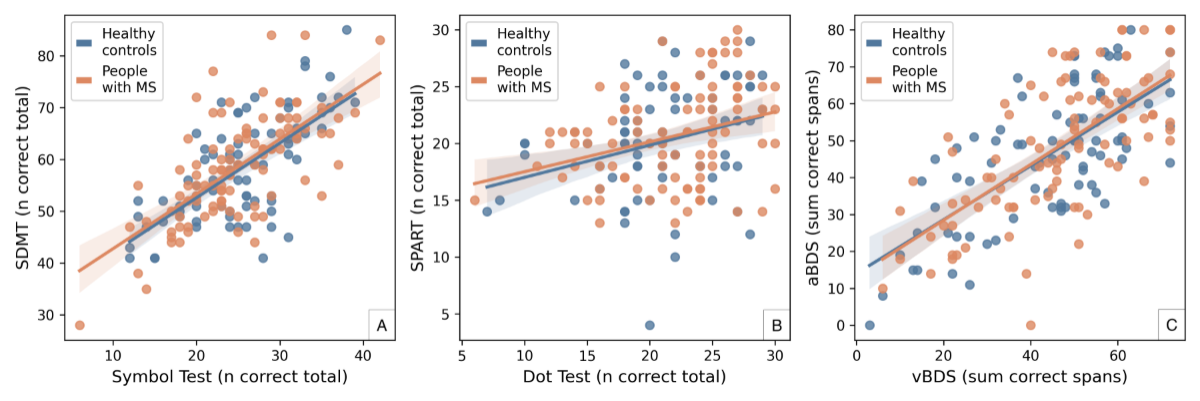
Concurrent validity. Scatterplots comparing the scores of each icognition test (x-axis) with those of its corresponding paper-and-pencil equivalent (y-axis). Correlations for both people with multiple sclerosis (MS) and healthy controls were (A) moderate to good, (B) fair, and (C) moderate to good. aBDS: auditory backward digit span; SDMT: Symbol Digit Modalities Test; SPART: 10/36 Spatial Recall Test; vBDS: visual Backward Digit Span.

Figure 2 shows the scatterplot of each icognition test with its paper-and-pencil equivalent. The Symbol Test showed a significant moderate to good correlation with SDMT performance (healthy controls: *r*=0.68; *P*<.001; people with MS: *r*=0.67; *P*<.001). There was also a significant fair correlation between the Dot Test and the SPART (healthy controls: *r*=0.30; *P*=.007; people with MS: *r*=0.31; *P*=.002) and a moderate to good correlation between the vBDS and its auditory equivalent (healthy controls: *r*=0.69; *P*<.001; people with MS: *r*=0.69; *P*<.001).

### Test-Retest Reliability

**Figure 3:**
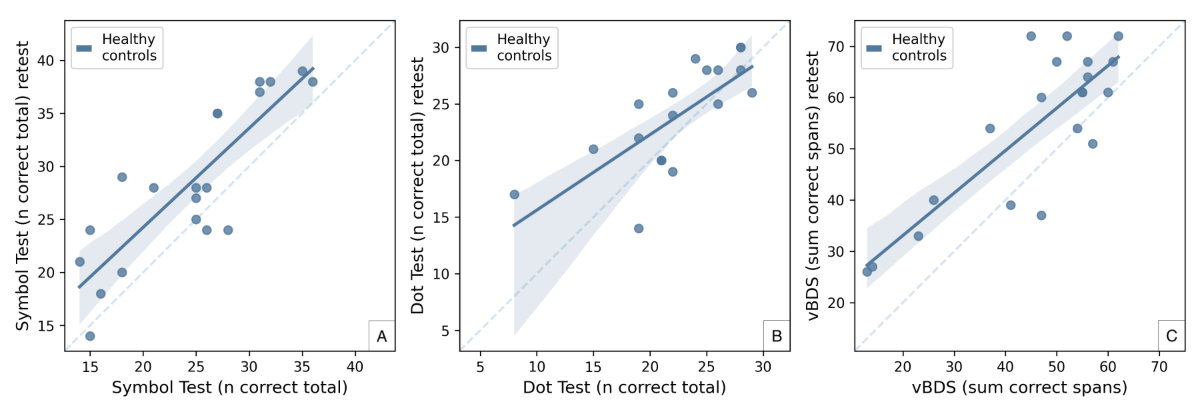
Test-retest reliability. Scatterplots comparing the scores of each icognition test at baseline (x-axis) with the retest scores an average of 18 (SD 3) days later (y-axis). All tests demonstrated moderate test-retest reliability. vBDS: visual Backward Digit Span.

In total, 20 healthy controls were retested with an average intertest interval of 18 (SD 3, range 14-23) days after initial testing to establish test-retest reliability. For the Dot Test, there was 1 missing value at baseline testing (n=19). Test-retest reliability (Figure 3) was moderate for the Symbol Test (ICC=0.74, *r*=0.85; *P*<.001), moderate for the Dot Test (ICC=0.71, *r*=0.74; *P*<.001), and moderate for the vBDS (ICC=0.72, *r*=0.83; *P*<.001).

### Difference Between People With MS and Healthy Controls

**Figure 4:**
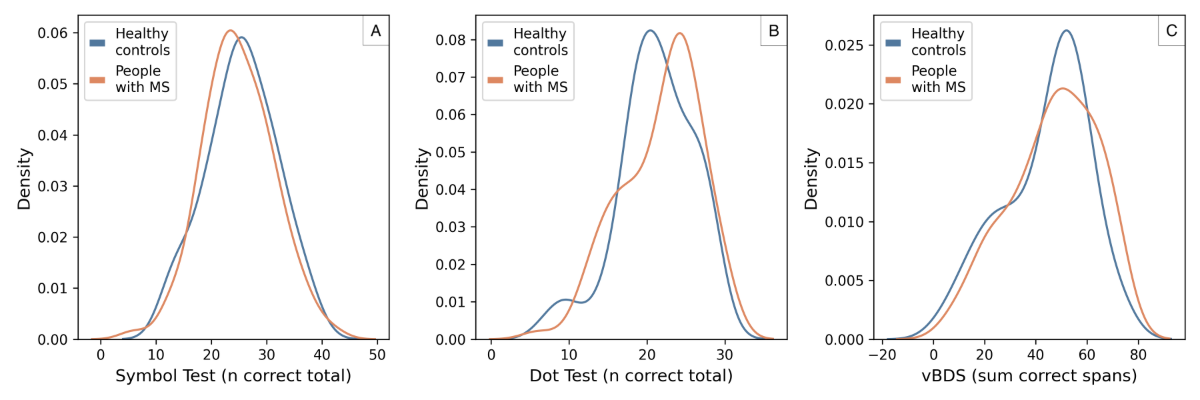
Comparison of the performance of healthy controls and people with multiple sclerosis (MS) on the icognition tests. Test performance was not significantly different between people with MS and healthy controls for all icognition tests. vBDS: visual Backward Digit Span.

For all icognition tests, there was no significant difference in performance between healthy controls and people with MS (Symbol Test: *U*=4431; *P*=.42; Dot Test: *U*=3516; *P*=.32; vBDS: *U*=3708; *P*=.27; Figure 4). The results were similar for all paper-and-pencil tests (SDMT: *U*=4060.5; *P*=.82; SPART: *U*=3934; *P*=.74; auditory Backward Digit Span: *U*=3824.5; *P*=.37; Figure S2 in Multimedia Appendix 1).

### Correlations With Clinical Parameters

A correlation matrix between the icognition tests and different clinical tests is presented in Table 2. In general, higher scores on the icognition tests were associated with less physical disability (measured using the EDSS), younger age, and a higher education level. Furthermore, all tests except the Symbol Test showed a correlation with disease duration, whereas fatigue (measured using the FSMC) was negatively associated with Dot Test performance. No correlation was found between any icognition test and depression (measured using the BDI). Correlation magnitudes were small or fair, with the exception of the moderate to good correlation between the Symbol Test and age (|*r*|=0.52).

**Table 2:** Correlation matrix of icognition tests with clinical variables. The Spearman correlation was used for the Beck Depression Inventory (BDI), Fatigue Scale for Motor and Cognitive Functions (FSMC), Expanded Disability Status Scale (EDSS), and education level, while the Pearson correlation was used for age and disease duration.

	Symbol Test		Dot Test		vBDS^a^	
	Correlation	*P* value	Correlation	*P* value	Correlation	*P* value
Age	−0.52	<.001	−0.29	.004	−0.27	.008
Education level	0.20	.04	0.37	<.001	0.22	.03
BDI	−0.03	.78	−0.12	.27	0.03	.80
FSMC	−0.10	.31	−0.26	.01	−0.03	.79
EDSS	−0.34	<.001	−0.32	.003	−0.21	.04
Disease duration	−0.17	.08	−0.28	.006	−0.31	.002

^a^vBDS: visual Backward Digit Span.

## Discussion

### Overview

In this paper, we present the results of the validation process of a smartphone-based screening battery for cognitive problems in people with MS. All tests correlated with their paper-and-pencil equivalents (concurrent validity), although correlation between the Dot Test and the SPART was only fair, most likely due to the fact that the SPART—the gold standard—lacks a distractor phase. The distractor phase was initially added to avoid ceiling effects and increase sensitivity in detecting cognitive decline. However, other test differences between the Dot Test and the SPART, such as grid size and the number of trials, could also cause the correlation to be weaker. Furthermore, all tests correlated with various clinical variables (age, education level, and EDSS) and showed moderate test-retest reliability. These findings indicate the suitability of the battery for routine remote screening of cognitive problems, a condition that many people with MS are likely to develop over time [8].

### State of the Art

We performed a systematic search (Multimedia Appendix 1) to identify smartphone-based cognitive tests for MS, which are summarized in Table 3. In brief, these apps began to emerge in 2020 and predominantly use a typical SDMT design. In this format, the mobile phone is held in landscape orientation, and a key of 9 symbol-digit pairs is displayed at the top. For 90 seconds, 1 symbol at a time is shown in the center of the screen, and the participant selects as quickly as possible the matching digit from 9 digit buttons located at the bottom of the screen. These tests are primarily designed to measure IPS, although they are also used to measure cognitive fatigability [39,40]. Other tests designed to measure specific cognitive domains include the smartphone version of the Trail Making Test Part B [41] for executive function and the Auditory Test of Processing Speed for IPS [42]. Furthermore, smartphone keystroke analysis [43,44] and various cognitive training games [45] were not designed for a specific cognitive domain, but their relationships with multiple cognitive domains was assessed. Recently, the first smartphone-based cognitive screening battery (DIGICOG-MS [15]) was introduced, marking an important next step in smartphone-based cognitive assessment.

**Table 3:** Summary of the state of the art in smartphone-based cognitive tests for people with multiple sclerosis (MS). The “Target domain” column indicates the specific cognitive domain assessed by each cognitive test.

Name	Summary	Target domain
MCT^a^ [46]	The MCT, part of MSCopilot, is a digital version of the SDMT^b^. In the MCT, a key displaying 9 symbol-digit pairs is presented at the top of the screen, with an exit button located in the top right corner. One symbol at a time is presented below the key. No details pertaining to the length of the test or scoring method are provided in the paper.	Information processing speed
sSDMT^c^ [35]	The sSDMT, part of MS Sherpa, is a digital version of the SDMT. In the sSDMT, a symbol-digit key (with 9 pairs) is presented at the top of the screen. A single symbol is then presented in the center of the screen, and participants must quickly tap the correct matching digit from 9 digit buttons presented at the bottom. The total score is the number of correct digits selected in 90 s. A timer is displayed, and the key is randomized at each trial.	Information processing speed
Voice-controlled DSST^d^ [47]	The voice-controlled DSST is part of the elevateMS app. Although limited details are provided, the app seems to be a variant of the SDMT, in which responses are collected via microphone.	Information processing speed and working memory
sSDMT [27]	The sSDMT, part of the Neurological Functional Test Suite, resembles the sSDMT (described previously). However, 3 differences are noteworthy. First, no timer is provided. Second, the test duration is 75 s; a 90-s performance score is calculated by multiplying the result by 90/75. Finally, the app also supports vocalized responses using a microphone and voice recognition to test participants with severe motor impairments.	Information processing speed
Mobile Trail Making Test Part B [41]	The app displays 13 circles, 6 containing a letter and 7 containing a number. The goal of the test is to connect the circles as quickly as possible in order, alternating between numbers and letters (1-A-2-B and so on, up to 7). The smartphone test was introduced by Ross et al [48] and assessed in people with MS in the study by Chen et al [41].	Executive function
Neurokeys [43]	Neurokeys is an alternative keyboard that records typing events (press and release) and subsequently calculates a range of features such as press duration and time interval between 2 presses.	No specific cognitive domain
BiAffect [44]	Similar to Neurokeys, the BiAffect app features a custom keyboard that temporarily replaces the smartphone’s default keyboard and is used to collect keystrokes and subsequently calculate various features.	No specific cognitive domain
cFAST^e^ [40]	The cFAST is similar to the sSDMT but includes an exit button and a progress bar to indicate the remaining response time. Unlike the 90-s duration of the sSDMT, the cFAST test lasts 5 minutes because the goal is to assess cognitive fatigability rather than information processing speed.	Cognitive fatigability
eSDMT^f^ [49]	The eSDMT, part of Floodlight, is an electronic version of the SDMT. Although few details are provided, the app seems to use a similar design to the sSDMT [39,50]. However, no timer or randomization of the key is mentioned.	Information processing speed
Cognitive games in the dreaMS app [45]	Several cognitive games were included in the dreaMS app featured in the study by Pless et al [45].	No specific cognitive domain
Auditory Test of Processing Speed [42]	Participants are presented auditory digits between 1 and 99 across 3 trials (20 digits each), with each trial involving different questions for the participants to answer (ie, trial 1: is the digit greater than 50? Trial 2: is the digit greater than 50 or odd? Trial 3: is the digit greater than 50 and odd?). The test examiner records the participants’ responses, and response time is calibrated before testing.	Information processing speed
MS Care Connect [51]	The MS Care Connect app is freely available for remote health assessments, including cognitive tests, in people with MS. Although limited information is available about the cognitive tests, a scoping review by Michaud et al [52] mentions it contains an SDMT-like test. According to a video on the app’s website, the test is administered in portrait orientation, with the key (9 symbol-digit combinations) at the top of the screen, a symbol presentation field below it, and a 3×3 answer keypad at the bottom [53].	Information processing speed
Konectom [54]	The test is designed to measure cognitive processing speed, with the smartphone held in portrait orientation. A symbol-digit key (with 9 pairs) is displayed at the top of the screen and remains fixed for the 90-s test duration. Symbols are presented in the center of the screen, and a 3×3 answer keyboard (digits 1-9) is located at the bottom. To correct for visuomotor interference, a separate test is included in which participants are asked to tap, in the same 3×3 answer keyboard and for 20 s, as many numbers as possible that are consecutively displayed in the center of the screen.	Information processing speed
DIGICOG-MS [15]	This is a smartphone-based cognitive battery with tests for memory, semantic fluency, and information processing speed. All tests seem to be designed for use in portrait orientation. A symbol-digit key (with 9 pairs) is presented at the top of the screen and remains fixed for the 90-s test duration. Four symbols are shown at a time in the center of the screen, and a 3×3 answer keyboard (digits 1-9) is located at the bottom.	Visuospatial memory, verbal memory, semantic fluency, and information processing speed

^a^MCT: Mobile Cognition Test.

^b^SDMT: Symbol Digit Modalities Test.

^c^sSDMT: smartphone Symbol Digit Modalities Test.

^d^DSST: Digit Symbol Substitution Test.

^e^cFAST: Cognitive Fatigability Assessment Test.

^f^eSDMT: electronic Symbol Digit Modalities Test.

The icognition screening battery differs from the state of the art in 2 ways. First, fine motor and visual problems are frequently reported in people with MS [55,56]. These symptoms are likely to interfere with time-based digital processing speed tests and could be affected by design considerations, as seen in the common digital SDMT design. This design includes multiple answer buttons (typically 9), and if configured in portrait orientation may result in a small symbol-digit key, especially on smaller devices. Although the problem is recognized by Woelfle et al [50] and the Scaramozza et al [54], the latter implementing a separate test to quantify motor interference, we opted to minimize motor and visual interference through design. Specifically, we used a simplistic design in landscape orientation, featuring 2 large answer buttons (Figure 1). Second, rather than a single test, icognition features a battery of tests designed to capture a broader cognitive profile because cognitive problems in people with MS are not limited to slowed IPS [14]. To the best of our knowledge, only Podda et al [15] have recently published a smartphone-based cognitive battery for people with MS. Moreover, icognition will be included in a digital care platform assessing symptoms, imaging biomarkers, and patient-reported outcomes [21] to provide a holistic picture of a patient’s well-being.

### A Cognitively Preserved Sample of People With MS

People with MS scored equally well on all tests compared to healthy controls. However, as can be observed in Table 1 and Figure S2 in Multimedia Appendix 1, this was also the case for the paper-and-pencil tests. Indeed, we seem to have included a sample of people with MS with relatively preserved cognition. We tested this in a post hoc analysis using the ANOVA method described by Anders [57], comparing the SDMT performance scores of our sample of people with MS (mean 58.5, SD 10.0; n=101; Table 1) with those reported in previous studies. López-Góngora et al [58] report an average SDMT performance score of 54.3 (SD 13.4; N=237), while Sousa et al [59] mention an average SDMT performance score of 53.51 (SD 11.76; n=115). We found that the people with MS included in this study performed significantly better (comparison with López-Góngora et al [58]: *F*_1,336_=8.01; *P*=.005; comparison with Sousa et al [59]: *F*_1,214_=11.1; *P*=.001).

The underlying reason for having selected a cognitively preserved sample is most likely the sampling bias resulting from the limited inclusion and exclusion criteria and the recruitment of patients during outpatient consultation in secondary and tertiary MS care settings. In these settings, participants are tested cognitively at least annually, which might enhance their familiarity with typical cognitive tests for MS, with a carryover effect to derivative tests, such as those in icognition. Moreover, people with MS who were able and willing to participate might (1) have better cognitive abilities, (2) be better at handling a smartphone, and (3) be familiar with scientific studies. The reason for being less strict on inclusion was to be able to offer the screening battery to any patient with MS who is regularly followed up in an outpatient setting. Future studies should confirm the validity of icognition in people with MS who are cognitively impaired. Moreover, combined with the publicly available data underlying this study, the sensitivity of icognition in detecting cognitive impairment could be established.

To investigate the impact of cognitive impairment on our results, we performed a post hoc analysis excluding participants who were considered impaired on at least 1 paper-and-pencil test (Figures S3-S5 and Table S3 in Multimedia Appendix 1). Compared to the original analysis, the most notable differences were observed in the correlations with clinical parameters, where the correlation between the Symbol Test and disease duration became significant (*P*=.04), while significance dropped for the relationship between the vBDS and education level (*P*=.06), the Dot Test and FSMC (*P*=.18), and the vBDS and EDSS (*P*=.20). Results for concurrent validity, test-retest reliability, and the difference between people with MS and healthy controls were comparable.

### The Benefits of Regular Digital Follow-Up

Proper and regular follow-up of cognitive function is important to capture fluctuations in cognitive state, such as those caused by a disease exacerbation or a relapse. Although Giedraitiene et al [60] show that a short cognitive screening tool such as the Brief International Cognitive Assessment for Multiple Sclerosis [18] can detect these cognitive fluctuations [60], the cognitive dip is likely missed in reality because cognitive assessments are usually performed only once or twice a year during clinical follow-up visits. The icognition screening battery allows for more frequent cognitive assessments. Furthermore, testing can be performed wherever and whenever a patient feels ready for it, reducing biasing effects such as fatigue [61]. Moreover, digitalization facilitates data collection and allows the extraction of more information from a cognitive test. As icognition associates every response with a 13-digit time stamp (with millisecond precision), cognitive fatigue or other performance metrics such as the “maximum gap time between correct responses” [39] can be tracked with high temporal resolution. For the Symbol Test, analogous examples are provided in the studies by Ganzetti et al [39] and Barrios et al [40].

Implementing icognition in a health care platform that includes fine motor assessment [62], symptom logging, and patient-reported outcome measures [21] enables patients to take an active role in their disease management; they can provide the individual information that complements the professional knowledge of the treating physician [63]. Moreover, collecting confounding variables, such as fatigue [21], at cognitive assessment improves the interpretation of cognitive scores.

### Limitations and Future Work

This study has some limitations. This validation study used a cross-sectional design, aside from the assessment of test-retest reliability. Therefore, we were unable to map the cognitive evolution of people with MS over time. These and other validated digital cognitive tests could considerably facilitate longitudinal testing in future studies, analogous to the 1-year follow-up study by Lam et al [64].

Popular screening batteries such as the Brief International Cognitive Assessment for Multiple Sclerosis also include a measure of verbal memory, which is indeed commonly impaired in MS [14]. However, we chose not to implement a digital test of verbal memory in icognition because such tests usually rely heavily on language, which complicates international use with the need for language-specific validation. Moreover, this introduces new technological difficulties, such as variations in microphone and speaker quality or the accuracy of speech recognition software. Although the aim of this study was to create a quick, home-based cognitive screening assessment, we acknowledge the importance of more in-depth multidomain cognitive assessment, including the assessment of verbal memory.

In the initial version of the Dot Test, the final 5 trials included remembering the position of the 3 dots on a 5×5 grid, but, after testing 3 participants with MS, we decided to consistently use a 4×4 grid, given the difficulty of the task, and excluded these participants from the Dot Test analyses. However, including these 3 participants in a post hoc analysis yielded similar results.

Furthermore, we tested consistently on the same Android smartphone (Samsung Galaxy A10) to avoid a biasing influence of model-specific factors, such as screen size, weight, and responsiveness, because these factors might impact performance and user experience. Although we expect our results to be applicable to other smartphone models, this limits the study findings to this smartphone model. However, as icognition has been designed using the Flutter framework (Google LLC), it can also be deployed on operating systems other than Android. An external validation study is planned to show the robustness of the app on different devices in a home setting.

To test concurrent validity, we used the SPART as the ground truth test for visuospatial memory because this closely resembled the Dot Memory Test presented in the study by Sliwinski et al [24]. Although recent evidence suggests that the SPART is among the most sensitive memory assessments in MS [65], Strober et al [66] found the Brief Visuospatial Memory Test–Revised to be more sensitive than the SPART. One might argue that the sole criterion of the ground truth test is that it measures visuospatial memory, regardless of its similarity to the digital test. In this context, using the Brief Visuospatial Memory Test–Revised would have been justified. Although the concurrent validity of the Dot Test was only fair, it demonstrated an acceptable test-retest reliability and correlated with several clinical variables, underscoring its value for clinical practice. Unlike the Symbol Test, which can be seen as a digital alternative to its paper-and-pencil reference test (SDMT), more research is necessary to establish the ground truth reference for the Dot Test. Furthermore, future research could subject icognition to a real-world (referred to later in this subsection), longitudinal study to establish its sensitivity to change at the individual level.

Test-retest reliability was assessed only in the healthy controls as per the recommendations of Benedict et al [28], who stated that test-retest reliability “can be investigated in a small sample of MS and/or healthy volunteers over 1-3 weeks.” Although we deem this to be a good indication for test-retest reliability in people with MS, substantiated by similar ICC reliability scores in the smartphone SDMT study by van Oirschot et al [35], test-retest reliability is not guaranteed to be similar for the cognitive tests in icognition. As we strove to perform test-retest reliability in similar circumstances (eg, location), assessing it for people with MS was difficult because they were tested during a 1-time visit to the outpatient MS clinics for consultation. Furthermore, we acknowledge that, although we followed the recommendations of Benedict et al [28], the sample sizes for testing test-retest reliability were small (Symbol Test and vBDS: n=20; Dot Test: n=19).

By recruiting a cognitively preserved sample of people with MS, this study was unfortunately unable to provide a realistic assessment of the sensitivity of the app to discriminate between (1) people with MS and healthy controls and (2) people with MS who are cognitively impaired and people with MS who are cognitively preserved. As the goal of icognition is not the diagnosis of MS but to screen for people with MS who are cognitively impaired, we especially recognize the latter as a limitation of this study. Indeed, of the 101 participants with MS, only 7 (6.9%), 8 (7.9%), and 9 (8.9%) presented impairment on the Symbol Test, Dot Test, and vBDS, respectively, resulting in a substantial class imbalance with respect to preserved test performance. However, because we make our data publicly available, this analysis can be conducted by future studies evaluating icognition on a sample of people with MS who are cognitively impaired.

This study validated the icognition app in controlled laboratory conditions under the supervision of a test examiner. However, we acknowledge that future studies should confirm psychometric properties such as test-retest reliability in remote, unsupervised settings. Furthermore, as recently demonstrated by Podda et al [15], the usability of cognitive apps should be assessed to assure a smooth transition to a real-life setting. A usability study is planned for icognition as well.

### Conclusions

This study established the reliability and validity of a newly developed smartphone app, icognition, for remotely screening cognitive impairment in terms of IPS and memory in people with MS. This allows for regular screening of cognitive performance to more quickly detect and respond to potential deterioration.

## Appendix

Multimedia Appendix 1 Supplementary text, tables, and figures.

## Abbreviations

BDI: Beck Depression Inventory
EDSS: Expanded Disability Status Scale
FSMC: Fatigue Scale for Motor and Cognitive Functions
ICC: intraclass correlation coefficient
IPS: information processing speed
MS: multiple sclerosis
SDMT: Symbol Digit Modalities Test
SPART: 10/36 Spatial Recall Test
vBDS: visual backward digit span

## Data Availability

The anonymized source data on which this paper relies, as well as the code used for statistical analysis and the creation of figures, are available in the GitHub repository of our laboratory [67].
