# Supplementary material for: A New Smartphone-Based Cognitive Screening Battery for Multiple Sclerosis (icognition): Validation Study
Source: J Med Internet Res. 2025 Jan 20;27:e53503. doi: 10.2196/53503 (PMC11791456; doi:10.2196/53503)
Supplement: Multimedia Appendix 1 [file jmir_v27i1e53503_app1.docx]

Supplementary material

# Digit spans

The digit spans used in the auditory Backwards Digit Span are listed in table S1.

| Span length | Digit span |
| --- | --- |
| 3 | 927 |
| 3 | 843 |
| 4 | 3164 |
| 4 | 5360 |
| 4 | 7918 |
| 4 | 4895 |
| 5 | 28452 |
| 5 | 26019 |
| 5 | 30475 |
| 5 | 84857 |
| 6 | 213604 |
| 6 | 918536 |
| 6 | 639271 |
| 6 | 728938 |
| 7 | 2395480 |
| 7 | 5837686 |

*Table S1: Digit spans used in the auditory backwards digit span*

# Z-normalisation

## Procedure

To normalise the ico**gnition** test scores for age, sex (male: 1, female: 2) and education level (years of education), for each ico**gnition** test, we fitted a linear regression equation with the test performance as dependent variable and age, sex and education level as independent variables. We then predicted the expected test score of HC and MS subjects given the healthy control regression equation, and subtracted the predicted value from the true test score, yielding the prediction error ($\varepsilon_{pred}$).

$${test\_score}_{pred}= w_{0}+w_{1}*sex+ w_{2}*education\_level+ w_{3}*age$$

(eq 1)

$$\varepsilon_{pred}={test\_score}_{norm}-{test\_score}_{pred}$$

(eq 2)

For each subject, the z score is the prediction error normalised with respect to the standard deviation of the prediction error distribution of the healthy control subjects.

$$z=\frac{\varepsilon_{pred}}{std(\varepsilon_{pred,HC})}$$

(eq 3)

## Necessary values

The necessary information to perform the procedure above is included below.

|  | Symbol Test | Dot Test | vBDS | SDMT | SPART 10/36 | aBDS |
| --- | --- | --- | --- | --- | --- | --- |
| w_0_ | 29.8987 | 28.8806 | 18.4677 | 54.9724 | 28.0566 | 5.7461 |
| w_1_ | 0.2170 | -1.5233 | 0.6054 | 1.0204 | -1.7338 | 3.8789 |
| w_2_ | 0.5795 | 0.1559 | 2.4597 | 1.0546 | 0.0740 | 3.0165 |
| w_3_ | -0.2849 | -0.1610 | -0.2506 | -0.2953 | -0.1308 | -0.2240 |
| std(ε_pred,HC_) | 4.4741 | 4.1522 | 15.2010 | 8.3418 | 4.0865 | 15.7066 |

*Table S2: Values for the normalisation procedure per ico****gnition*** *test (first three columns) and paper-pencil cognitive test (last three columns).*

## Test performance MS versus HC: normalized test scores

Figure S1 displays the performance of each ico**gnition** test after correcting test performance for expected performance from the healthy control dataset. People with MS and healthy controls (HC) had comparable test scores (Symbol Test (Z): U = 4730, p = 0.10; Dot Test (Z): U = 3815, p = 0.656; vBDS (Z): U = 3791, p = 0.383).


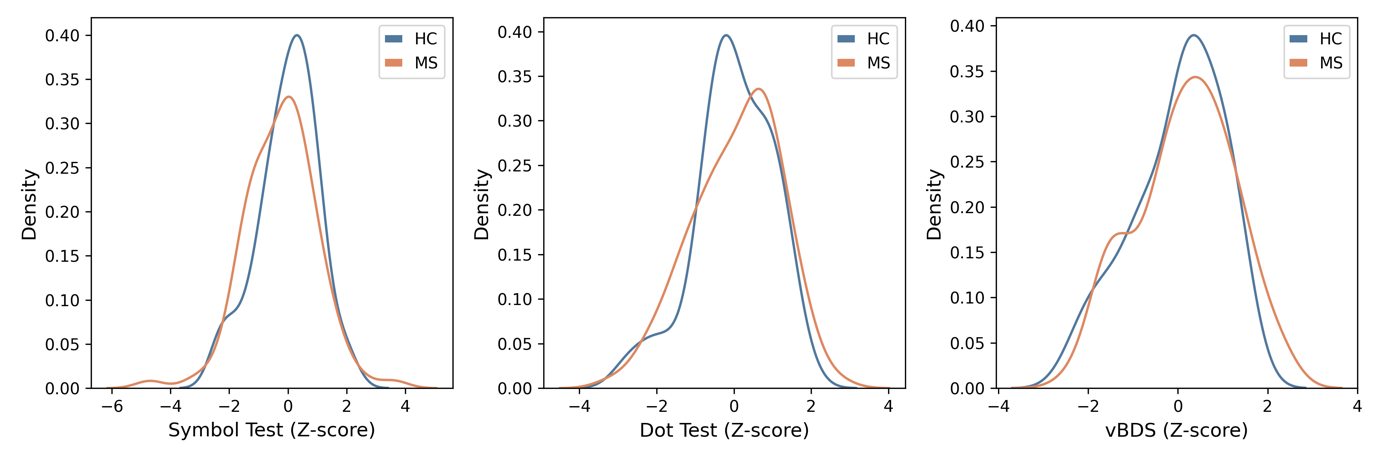


*Figure S1: Performance on normalized test scores of persons with MS and healthy controls. Test performance was not significantly different between MS and HC for all ico****gnition*** *tests.*

# Test performance MS versus HC: paper-pencil tests

None of the paper-pencil tests were significantly different between people with MS and HC subjects (SDMT: U = 4060.5, p = 0.82; SPART 10/36: U = 3934, p = 0.74; auditory Digit Span Backwards (aBDS): U = 3824.5, p = 0.37). The criterion validity (MS versus HC) for all paper-pencil test equivalents of all ico**gnition** tests can be consulted in figure S2.


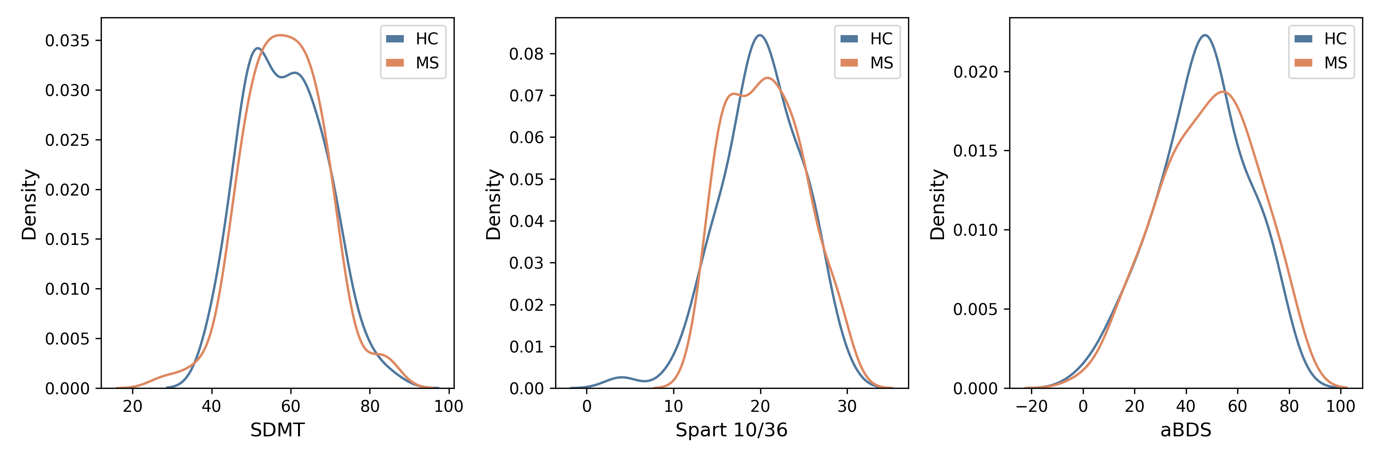


*Figure S2: Performance on paper-pencil tests of persons with MS and healthy controls. Test performance was not significantly different between MS and HC for all paper-pencil tests.*

# Systematic search strategy for state of the art

The Pubmed database was searched on 02/10/2024 using the following search string:

(((((((((cell phone[MeSH Terms]) OR (cell phones[MeSH Terms])) OR (cell* phone*[Title/Abstract])) OR ((smartphone*[Title/Abstract]) OR (smart* phone*[Title/Abstract]))) OR (mobile*[Title/Abstract])) OR ((iphone*[Title/Abstract]) OR (android*[Title/Abstract]))) OR ((telephone*[Title/Abstract]) OR (tele* phone*[Title/Abstract]))) OR (phone*[Title/Abstract])) AND ((cognit*[Title/Abstract]) OR (cognition[MeSH Terms]))) AND ((multiple sclerosis[MeSH Terms]) OR (multiple sclerosis[Title/Abstract]))

Number of hits: 179

# Post-hoc analysis without cognitively impaired subjects

This section contains the results of rerunning part of the analyses on subjects that did not have an impaired test performance on any of the paper-pencil tests, i.e., a z-score higher than -1.5.

**Concurrent validity:**

Figure S3 shows the scatterplot of each ico**gnition** test with its paper-pencil equivalent for cognitively preserved subjects. The Symbol Test significantly correlated moderate to good with SDMT performance (HC: r = .79, *P* < .001; MS: r = .64, *P* < .001). There was also a significant, fair correlation between the Dot Test and the SPART (HC: r = .37, *P* = .003; MS: r = .38, *P* < .001) and a moderate to good correlation between the vBDS and its auditory equivalent (HC: r = .54, *P* < .001; MS: r = .67, *P* < .001).

*
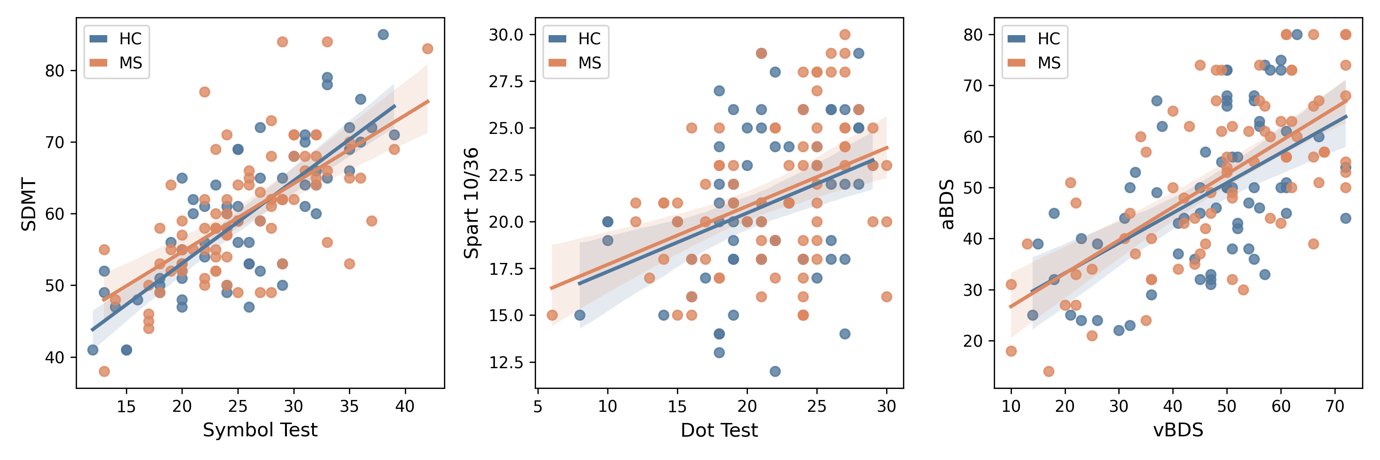
*

*Figure S3: Concurrent validity. Scatterplot of each ico****gnition*** *test (x-axis) and its paper-pencil equivalent (y-axis). Correlations for both MS and HC were moderate to good (left), fair (middle) and moderate to good (right). Data of cognitively preserved subjects.*

**Test-retest reliability:**

Test-retest reliability for cognitively preserved subjects (figure S4) was moderate for the symbol Test (ICC = .75, r = .88, *P* < .001, n = 17), moderate for the Dot Test: (ICC = .71, r = .74, *P* = .001, n = 16) and moderate for the vBDS (ICC = .65, r = .76, *P* < .001, n = 17).


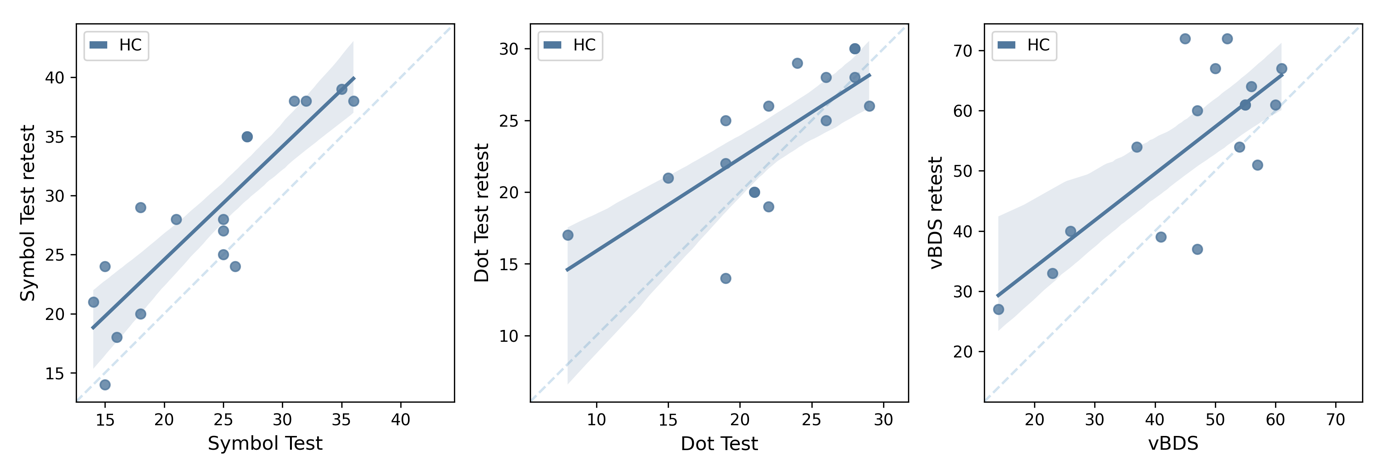


*Figure S4: Test-retest reliability between each ico****gnition*** *test at baseline (x-axis) and an average of 18 days later (y-axis). All tests had moderate test-retest reliability. Data of cognitively preserved subjects.*

**Performance HC vs MS:**

For cognitively preserved subjects. for all tests in ico**gnition**, there was no significant difference in performance between healthy subjects and people with MS (figure S5). Symbol Test: U = 2784.5, *P* = .89; Dot Test: U = 2371, *P* = .35; vBDS: U = 2402, *P* = .19.

*
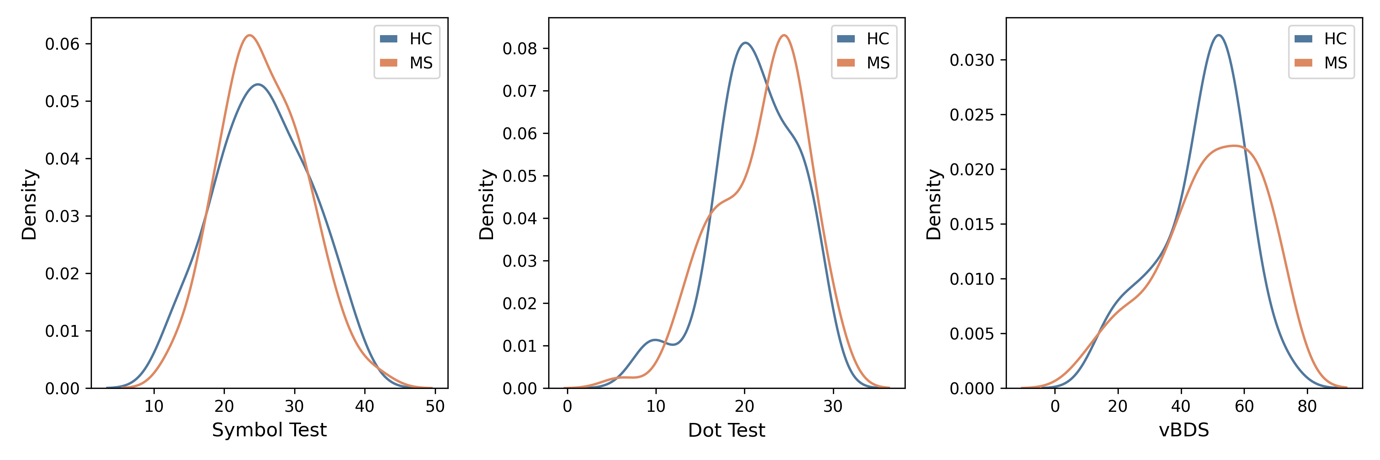
*

*Figure S5: Comparison of the performance of healthy subjects and people with MS on the ico****gnition*** *tests. Test performance was not significantly different between cognitively preserved MS and HC for all ico****gnition*** *tests. Data of cognitively preserved subjects.*

**Correlation with clinical parameters:**

|  | Symbol Test | Dot Test | vBDS |
| --- | --- | --- | --- |
| Age | -.55 (<.001) | -.28 (.01) | -.26 (.02) |
| Education level | .24 (.03) | .40 (<.001) | .21 (.06) |
| BDI | .03 (.82) | -.11 (.35) | .01 (.93) |
| FSMC | -.06 (.58) | -.15 (.18) | .02 (.89) |
| EDSS | -.35 (.002) | -.26 (.03) | -.15 (.20) |
| Disease duration | -.23 (.04) | -.24 (.04) | -.31 (.005) |

*Table S3: Correlation matrix of each ico****gnition*** *test with several clinical variables. Each value is represented as correlation (P-value). Spearman correlation was used for the BDI, EDSS, FSMC and education level, whereas Pearson correlation was used otherwise. Data of cognitively preserved subjects.*
